# The Role of Dynamic Neuromuscular Stabilization Exercises in Stress Urinary Incontinence Among Females Aged 18-40 Years

**DOI:** 10.7759/cureus.59828

**Published:** 2024-05-07

**Authors:** Kiran Sharma, Meena Gupta, Raju K Parasher

**Affiliations:** 1 Physiotherapy, Amity Institute of Health Allied Sciences, Amity University, Noida, IND; 2 Physiotherapy, Venkateshwar Hospital, University of Delhi, New Delhi, IND

**Keywords:** women, integrated spinal stabilization system, kegel exercises, stress urinary incontinence, dynamic neuromuscular stabilization

## Abstract

Background and objective: Stress urinary incontinence (SUI) is prevalent among females across various age groups, yet societal taboos and unawareness contribute to under-reporting and hinder effective management strategies. This study aimed to evaluate the efficacy of dynamic neuromuscular stabilization (DNS) compared to traditional Kegel exercises in females with stress urinary incontinence, focusing on assessing the impact of DNS on pelvic floor strength and core musculature activation to provide valuable insights into urinary continence management.

Methodology: This is a single-blinded, randomized trial with 90 females aged 18-40 years assessed perineometer readings, pelvic floor electromyography (EMG), and transverse abdominis activation via pressure biofeedback.

Results: Significant improvements in pelvic floor strength and core musculature activation were observed in the DNS group compared to the Kegel exercise group. Perineometer values, EMG measurements, and pressure biofeedback unit readings demonstrated substantial enhancements post-intervention in both groups. Effect sizes, including Cohen's D and point biserial correlation coefficient, indicated medium to large effects favoring the DNS intervention.

Conclusion: DNS is superior to Kegel exercises for SUI management, emphasizing the importance of targeting core musculature. Future research should explore long-term outcomes and patient-reported measures for a comprehensive understanding.

## Introduction

Urinary incontinence, specifically stress urinary incontinence (SUI), is a prevalent and distressing condition affecting individuals, predominantly females, across diverse age groups, with a reported global prevalence rate ranging from 4% to 35% [1]. This condition manifests as involuntary leakage of urine during activities that increase intra-abdominal pressure, such as sneezing, coughing, running, or lifting heavy objects [2]. Beyond its physical symptoms, SUI significantly disrupts an individual's social life and personal well-being, ultimately compromising their overall quality of life [3].

Despite the widespread occurrence of SUI, particularly in countries like India, where cultural norms may contribute to females enduring the condition silently, the reported prevalence rates might not accurately reflect the true scope of the problem [3]. A substantial portion of affected individuals remains hesitant to seek help due to reasons such as unawareness and societal taboos, leading to underreported statistics [3].

Stress urinary incontinence can arise from various underlying factors contributing to the impairment of urinary control mechanisms. One common cause involves deficiencies in the urethral closure system, often linked to pelvic surgeries, menopausal changes, or conditions such as urogenital atrophy. These factors can disrupt the ability of the urethra to maintain closure during activities that increase intra-abdominal pressure, leading to urine leakage [4].

Additionally, damage to the urethral support system plays a significant role in the development of stress urinary incontinence. This damage is frequently attributed to the effects of vaginal deliveries on the structures of the pelvic floor. The trauma from childbirth can result in altered nerve supply, injuries to the levator ani muscles, and weakening or damage to the endopelvic fascia. These structural changes can compromise the stability and support of the urethra, contributing to urinary leakage during activities such as sneezing, coughing, or physical exertion [4]. The integrated continence system, involving the intrinsic urethral closure system, urethral support system, and lumbopelvic stability system, plays a crucial role in maintaining urinary continence [4]. This intricate system relies on the coordination of various muscle groups in the lumbopelvic region, emphasizing the significance of a holistic approach to address SUI [4].

Contrary to a conventional focus solely on pelvic floor muscle strengthening, emerging evidence underscores the importance of other core muscles including transverse abdominis, diaphragm, and multifidus in achieving optimal results [5]. Pelvic floor muscle rehabilitation is posited to reach its full potential when addressing the entire core musculature concurrently [5]. The bidirectional relationship between pelvic floor and other core muscles emphasizes the potential benefits of integrating transverse abdominis, diaphragm, and multifidus muscle training into stress urinary incontinence (SUI) rehabilitation. It is noteworthy that the respiratory centers play a pivotal role in managing abdominal and pelvic muscles, while voluntary contraction is facilitated via spinal and sacral nerves [5]. Acknowledging these mechanisms underscores the importance of a comprehensive approach to SUI management, encompassing not only pelvic floor exercises but also respiratory and spinal muscle training.

To address the multifaceted nature of SUI comprehensively, the study introduces a promising approach known as dynamic neuromuscular stabilization (DNS) [6,7]. Grounded in developmental kinesiology, DNS compares stabilizing patterns to those of healthy infants, targeting the integrated spinal stabilization system involving deep cervical flexors, diaphragm, transversus abdominis, multifidus, and the pelvic floor [8]. DNS exercises aim to activate the spinal stabilizing system effectively through repetition, aiding individuals in regaining control during various tasks [6,8].

Dynamic core stability, vital for optimal athletic performance, relies on the coordinated action of the integrated spinal stabilization system and the regulation of intra-abdominal pressure [6,9]. DNS acknowledges the cohesive functioning of the core musculature, emphasizing the importance of addressing weak or dysfunctional components to prevent repercussions on the entire system [6,10,11]. DNS exercises are specifically designed to foster comprehensive activation and strengthening of the entire core musculature, including the diaphragm. It is important to note that the diaphragm muscle plays a crucial role in stabilizing the abdominal, lumbar, and pelvic areas. A correct breathing pattern ensures proper stabilization of the spine. By enhancing stability and support to the spine, pelvis, and surrounding structures, DNS exercises contribute significantly to addressing the multifaceted aspects of stress urinary incontinence (SUI). This acknowledgment underscores the holistic approach of DNS in promoting core stability and urinary continence [6,11].

While DNS has demonstrated effectiveness in diverse conditions, such as sports injuries, cerebral palsy, and hemiplegia, its impact on stress urinary incontinence (SUI) remains unexplored [9,11-14]. This study aimed to bridge this gap by evaluating the effectiveness of a dynamic neuromuscular stabilization exercise program in comparison to a traditional pelvic floor exercise program for females dealing with stress urinary incontinence. The intricate connections between the pelvic floor and core musculature underscore the necessity of a comprehensive approach to treating SUI, and this research seeks to contribute valuable insights in this domain.

## Materials and methods

The research project commenced subsequent to receiving approval from the Institutional Review Board of Amity University, Noida, India, and Amar Jyoti Institute of Physiotherapy, University of Delhi, New Delhi, India with IEC document number AUUP/IEC/2021-Jan/03 and AJ-IRB/22/2020, respectively. This study was registered in the Clinical Trial Registry - India under the reference CTRI/2021/09/036247. This study, structured as a single-blinded, single-center randomized controlled trial, enlisted 90 female participants through convenient sampling. G*Power software (Düsseldorf, Germany: G*Power Team, University of Düsseldorf), employing a t test, an effect size of 0.3, alpha error of 0.05, and a power of 0.8, determined the sample size. The participants were stratified into two following groups: the dynamic neuromuscular stabilization (DNS) group and the control group (Kegel exercise group), each comprising 45 individuals [7].

Eligible participants were females aged 18-40 years, married, diagnosed with mild-to-moderate stress urinary incontinence (SUI), at least one year post-delivery, and medically and physically fit for assessment and physiotherapy. Exclusion criteria comprised continuous urinary leakage, current UI drug therapy, pelvic prolapse > stage I, pregnancy, vaginal/urinary tract infections, menstruation during examination, presence of tumors/fractures/acute inflammatory diseases, current estrogen treatment, and use of anticholinergics, antidepressants, or serotonin-affecting substances [7]. Recruitment was facilitated by referrals from gynecologists. All the participants were diagnosed by the gynecologist.

Randomization and allocation of participants were accomplished at a 1:1 ratio using a random number table generated by a statistician. The allocation sequence was documented and sealed in opaque envelopes, with an independent individual responsible for revealing the group assignment. This process ensured a fair distribution of participants into either the DNS or Kegel exercise group.

**Figure 1:**
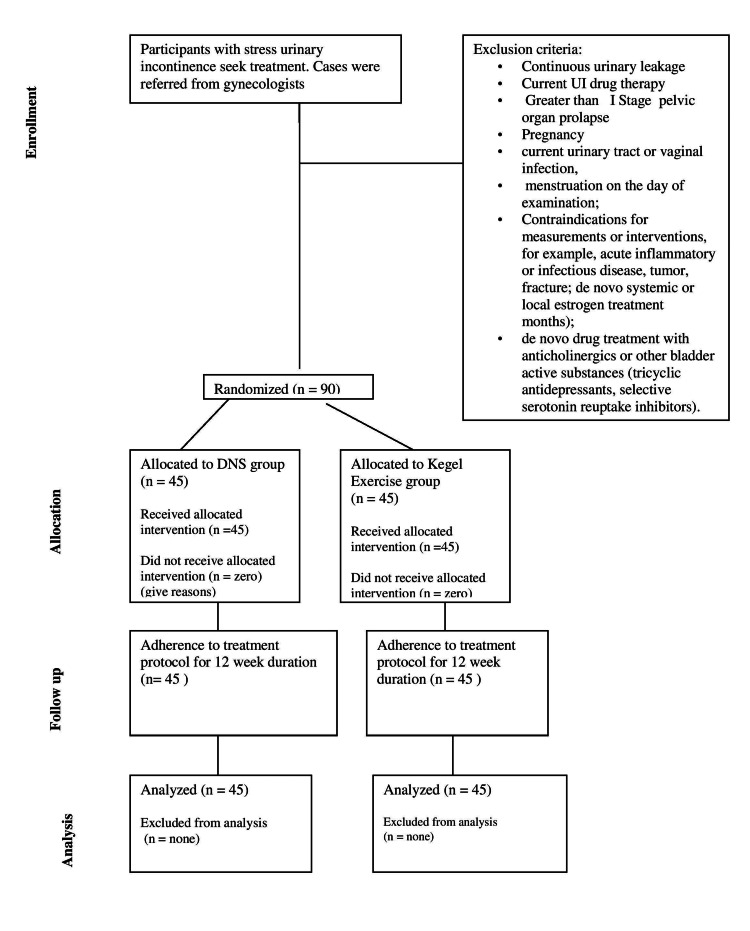
CONSORT flow chart for trial design. CONSORT: Consolidated Standards of Reporting Trials

Baseline measurements, encompassing pelvic floor muscle strength (perineometer), EMG of pelvic floor muscles, and transverse abdominis activation (pressure biofeedback) were conducted. These measurements were repeated after a 12-week intervention period. The Consolidated Standards of Reporting Trials (CONSORT) flowchart visually depicted participant enrollment, progression, inclusion, allocation, follow-up, and analysis stages (Figure 1).

The experimental group adhered to a four-phase exercise protocol outlined in Table 1, while the control group performed pelvic floor muscle contractions. The latter involved holding each contraction for 10 seconds, with 10 repetitions per set and three sets in total, separated by 1 minute of rest [15]. Both groups performed exercises in clinical settings under the supervision of the primary investigator.

**Table 1:** Exercise protocol for the rehabilitation of stress urinary incontinence using dynamic neuromuscular stabilization.

Phase	Duration (weeks)	Patient’s position	Investigator’s position	Instructions	Dosage
I	3	1. Crook lying	Palpating the transverse abdominis muscle on the patient's side, specifically 2 cm medial to the anterior superior iliac spine in the anatomical projection of the deep abdominal muscles	Engage the pelvic floor by gently contracting it, as if holding urine. While maintaining this contraction, inhale to allow your abdominal wall to expand against the therapist's fingers. Sustain this expansion as you exhale, then resume normal breathing	Frequency - 3 times per week; intensity - 3 sets of 10 repetitions; duration - 12 weeks; rest between 2 sets - 1 minute; contraction time for each contraction - 10 seconds; rest after each contraction - 10 seconds
		2. Prone	The area on the abdominal wall located posteriorly and laterally beneath the lower ribs when viewed from behind	Same as the previous exercise	
IIa	1.5	1. Supine with legs supported on a stool	Palpating the transverse abdominis muscle on the patient's side, specifically 2 cm medial to the anterior superior iliac spine	Same as phase I	
		2. Quadruped	The area on the abdominal wall located posteriorly and laterally beneath the lower ribs when viewed from behind	Same as phase I	
IIb	1.5	1. Supine with legs supported on gym ball	Palpating the transverse abdominis muscle on the patient's side, specifically 2 cm medial to the anterior superior iliac spine	Same as phase I	
		2. Quadruped with knees supported on an unstable surface	The area on the abdominal wall located posteriorly and laterally beneath the lower ribs when viewed from behind	Same as phase I	
III	3	Quadruped to heel sitting	Positioned at the rear of the patient	Instruct the patient to assume a heel sitting position, ensuring a neutral spine and engaging the abdominal and pelvic floor muscles. During the first phase, the therapist will provide resistance against the patient's contraction. In the second phase, the patient will attempt heel sitting while the therapist's force prevails, causing movement in the cranial direction	
IV	3	1. Sagittal stabilization with hip abduction	Palpating the transverse abdominis muscle on the patient's side, specifically 2 cm medial to the anterior superior iliac spine	Activate the pelvic floor slightly, as if holding urine. While sustaining this contraction, inhale to allow the abdominal wall to expand against the therapist's fingers. Maintain this expansion, exhale, and return to normal breathing. While sustaining normal breathing, instruct the patient to abduct one hip while keeping the other hip in the neutral starting position. Repeat this sequence on the opposite side	
		2. Sagittal stabilization with hip flexion	Palpating the abdominal muscle 2 cm medial to anterior superior iliac spine	Engage the pelvic floor by contracting it slightly, simulating the action of holding urine. While sustaining this contraction, inhale to allow the abdominal wall to expand against the therapist's fingers. Maintain this expansion as you exhale, then resume normal breathing. Throughout, instruct the patient to flex one hip while keeping the other hip in the neutral starting position. Repeat this sequence on the opposite side	

Outcome measures after a 12-week treatment period included changes in perineometer values, maximum voluntary contraction of pelvic floor muscles (PFM) through electromyography (EMG), and activation of the transverse abdominis muscle using pressure biofeedback. The apparatus used in the study (Mumbai, India: Bionics Perineometer Analogue) comprised a conical vaginal insert connected to a handheld microprocessor, facilitating the measurement of pressure in mmHg upon compression of the insert. Participants assumed a crook-lying position, and the perineometer was carefully inserted into the vaginal canal until the compressible portion extended beyond the hymeneal ring. Baseline pressure readings were recorded, and participants were then instructed to exert their PFM maximally for two to three seconds, completing three consecutive squeezes with a one-minute rest interval. The highest point reached during these three contractions represented their maximum perceived strength [16].

To ensure accuracy and prevent elevated perineometer readings caused by excessive intra-abdominal pressure, meticulous observations and a pressure biofeedback device were employed to maintain a neutral spine position. The vaginal insert was covered with a condom to ensure safe and hygienic use for multiple participants. The perineometer's high reliability was evident, as indicated by an intra-class correlation coefficient (ICC) of 0.95 [16].

The NeuroTrac MyoPlus2A version 11.1 device (Surrey, UK: Verity Medical Ltd.) was utilized to collect EMG data. A pear-shaped intra-vaginal sensor equipped with stainless steel electrodes was gently introduced into the vagina, with patients in a supine lithotomy position. A reference electrode was strategically placed on the right anterior superior iliac spine. Before the examination, participants underwent training to ensure precise contraction of their PFM. PFM strength was assessed by recording the average scores from three maximum contractions. The EMG values were documented for average (EMG average), peak (EMG peak), and maximum voluntary contractions (EMG MVC), with measurements in microvolts for EMG average and EMG peak (µV) and in percentage (%) for EMG MVC. An automated protocol software provided on-screen instructions and voice guidance, indicating the timing for contraction and relaxation of the PFM, following a pattern of five-second work and rest intervals [17]. The displayed data were obtained after filtration by the built-in software.

The Stabilizer Pressure Biofeedback Unit manufactured by Chattanooga Group Inc. (Vista, CA) was utilized. Participants assumed a prone position on a plinth with arms at their sides and their heads comfortably supported in a designated mold to maintain a straight and relaxed neck alignment. The pressure biofeedback unit was positioned beneath the lower abdomen, aligned with the anterior superior iliac spine [18].

Initially, the unit's bulb was inflated to a pressure of 70 mmHg. Participants were instructed to completely relax their entire body, particularly their abdomen, before each contraction. The objective was to assess participants' ability to perform abdominal hollowing by maintaining the contraction for four seconds within a ten-second interval, monitored using a digital watch. Participants were required to execute the abdominal hollowing action without resorting to substitute maneuvers, such as engaging the overall abdominal muscles or making evasive movements involving the spine, hips, pelvis, or shoulder girdle. Additionally, participants maintained regular, uninterrupted breathing [18].

The gauge displayed the overall change in pressure, recorded in millimeters of mercury (mmHg). The observer positioned both hands as recommended, medially and inferiorly to the anterior superior iliac spines, and laterally to the rectus abdominis muscle, to detect appropriate muscle contraction. Meeting the criteria for observation and palpation, as well as displaying a consistent decrease in pressure of at least 1 mmHg for four consecutive seconds within the 10-s timeframe (pressure criterion), indicated a positive outcome for the PRONE test [18].

Statistical analyses adhered to the Enhancing the Quality and Transparency of Health Research (EQUATOR) Network guidelines. Normal distribution was assessed using the Kolmogorov-Smirnov (KS) test, and non-normal data were transformed. Paired t tests assessed within-group differences, and unpaired t tests explored differences between the DNS and Kegel exercise groups. Mann-Whitney U test and Wilcoxon sign rank test were employed for non-normal variables.

Furthermore, Cohen’s D was computed in our investigation to gauge the effect size, where 0.2 is considered small, 0.5 medium, and 0.8 large. If the disparity between group means is less than 0.2 standard deviations, it is considered negligible, even in the presence of statistical significance. A substantial Cohen's D implies a significant and meaningful effect. While a p-value informs us of the existence of a significant difference between groups, the effect size provides a quantitative measure of the magnitude of that difference [19].

The formula for Cohen's D is expressed as follows: D = (M1 - M2)/S, where D represents Cohen's D (the effect size), M1 is the mean of the first group, M2 is the mean of the second group, and S is the pooled standard deviation [19]. Calculating Cohen's D was essential for assessing the magnitude of the difference between the groups receiving DNS and traditional Kegel exercises. This statistical measure helps interpret the practical significance of our findings beyond mere statistical significance.

However, point biserial correlation coefficient “r” was employed in case of non-parametric tests. The calculation formula used for "r" was expressed as follows: r = Z / √N, wherein r denotes the rank biserial correlation coefficient and Z signifies the z-score derived from the Mann-Whitney U test. The z-score, in this context, quantifies the number of standard deviations that an observation or data point deviates from the mean of a distribution. Meanwhile, √N corresponds to the square root of the total sample size [20]. A biostatistician, blinded to group assignments, conducted all analyses using SPSS version 23 software.

## Results

**Figure 2:**
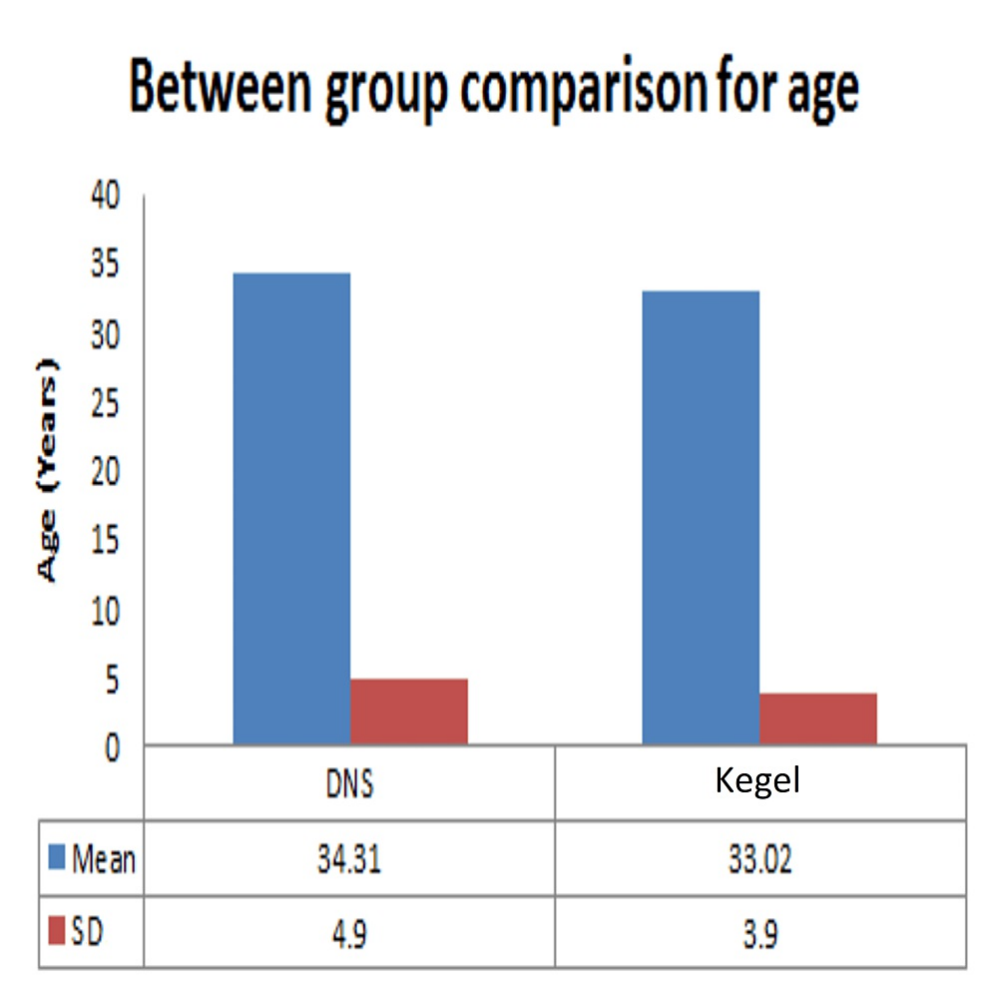
Comparison between the DNS and Kegel exercise group for age of the participants. Mean and SD of the age (years) are represented. No statistically significant difference was found between the age of two groups as p-value was >0.05. DNS: dynamic neuromuscular stabilization

**Figure 3:**
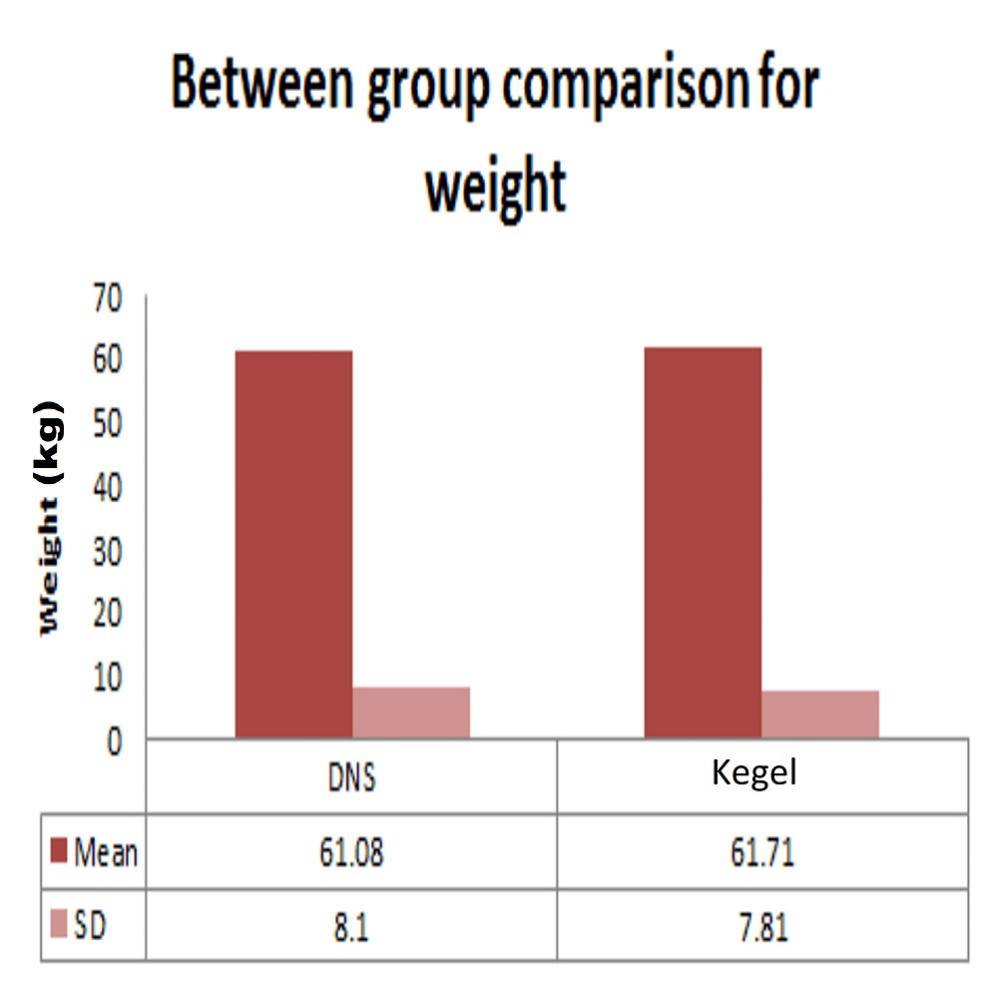
Comparison between DNS and Kegel exercise group for weight of the participants. Mean and SD of the weight of the participants in the two groups are given. No statistically significant difference was found between the weight of the participants of the two groups as p-value >0.05. DNS: dynamic neuromuscular stabilization

**Figure 4:**
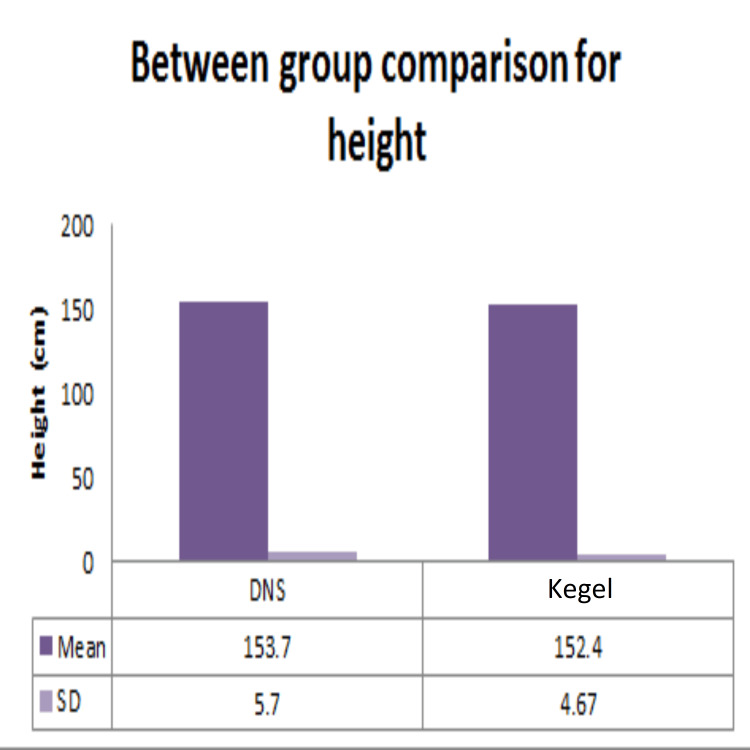
Comparison of height of participants in DNS and Kegel exercise group. Mean and SD of height of participants in the two groups. No statistically significant difference was found between the two groups as p-value >0.05. DNS: dynamic neuromuscular stabilization

**Figure 5:**
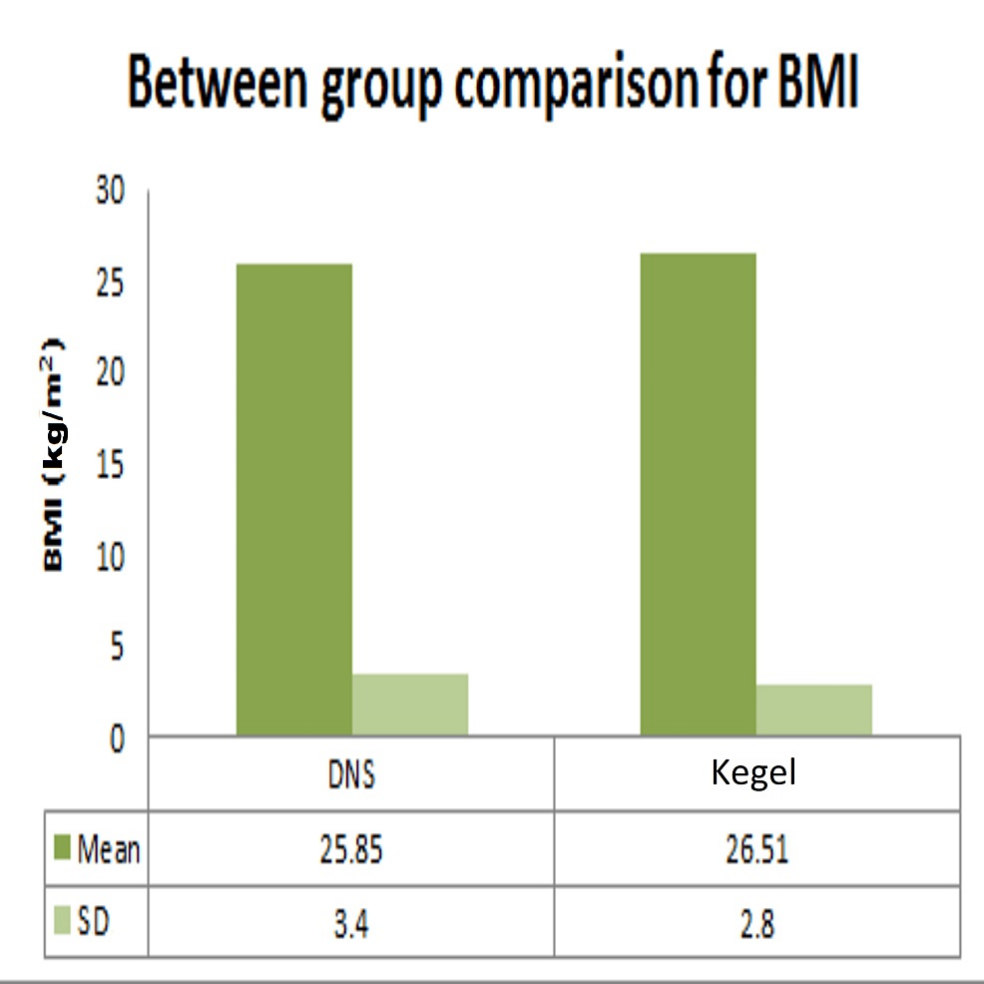
Comparison between DNS and Kegel exercise group for BMI. Mean and SD of BMI of the two groups. BMI: body mass index; DNS: dynamic neuromuscular stabilization

**Figure 6:**
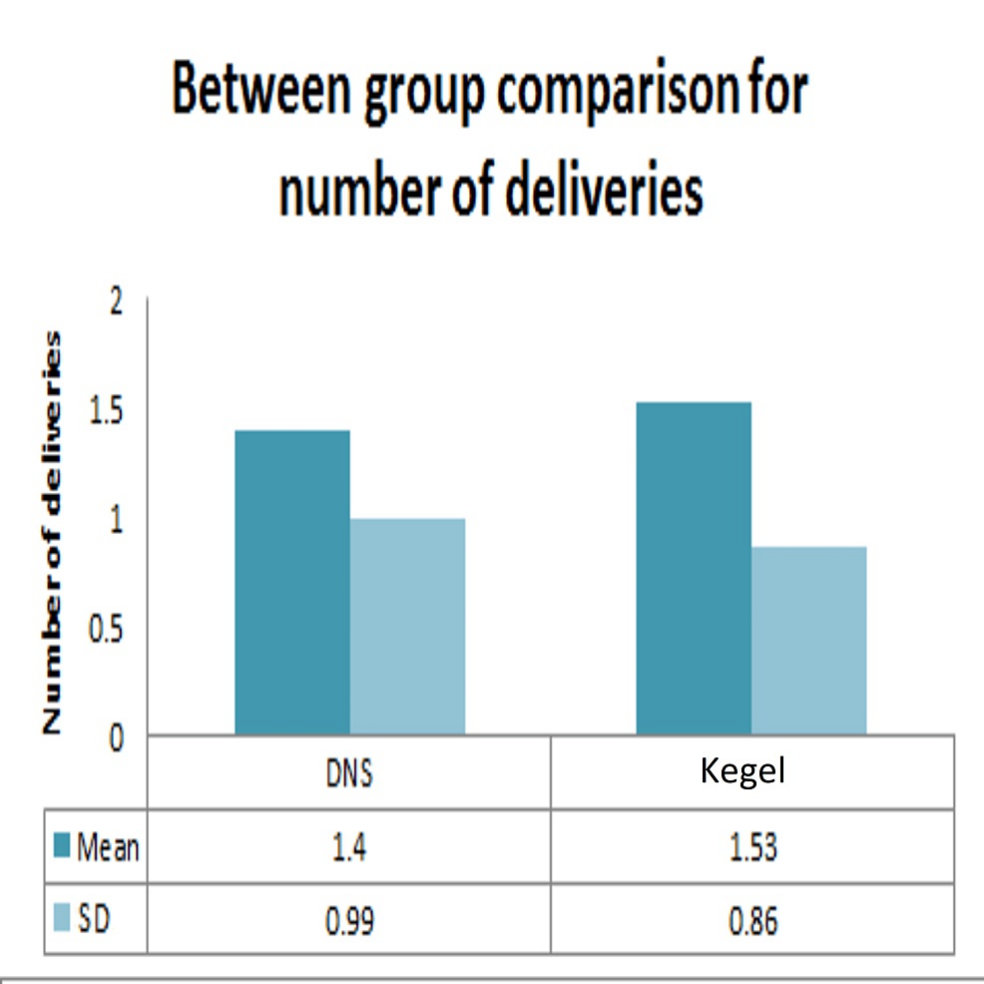
Comparison between DNS and Kegel exercise group for number of deliveries. Mean and SD of number of deliveries of the two groups. No statistically significant difference was found between the two groups as p-value >0.05. DNS: dynamic neuromuscular stabilization

**Figure 7:**
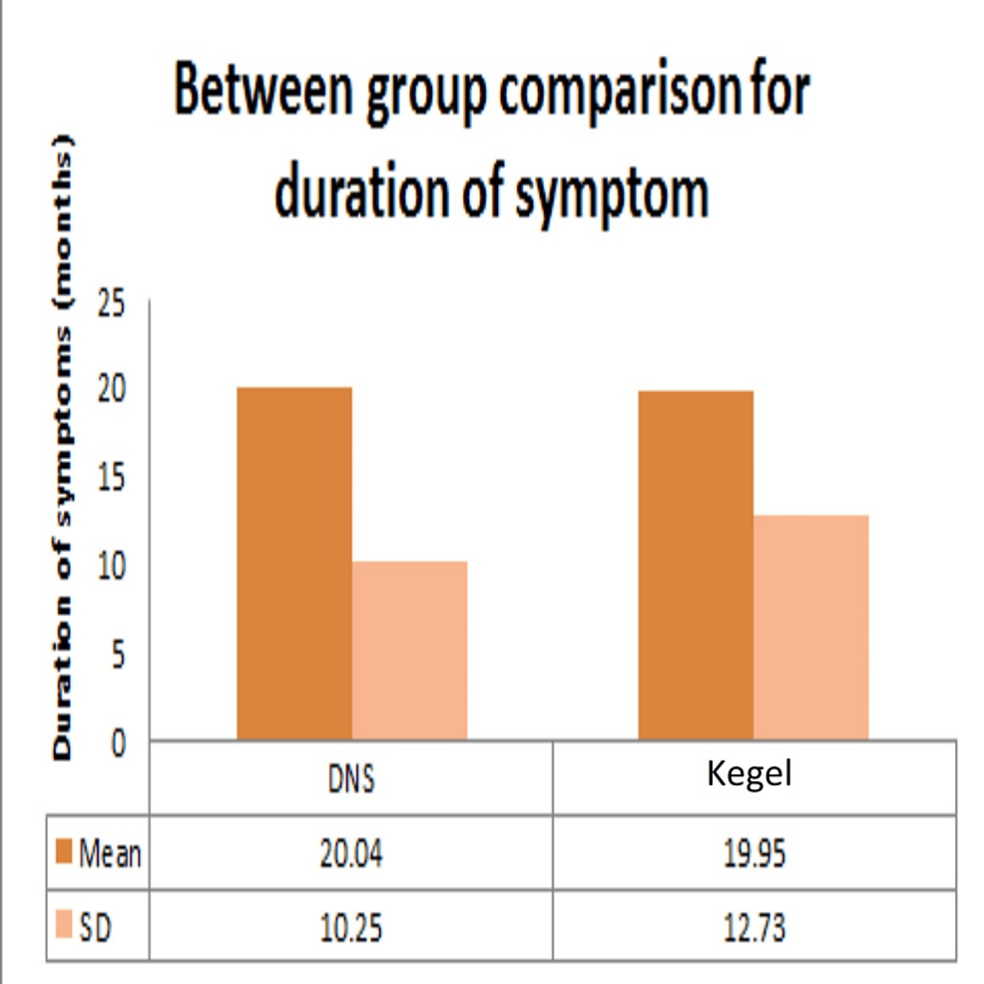
Comparison between DNS and Kegel exercise group for duration of symptom. Mean and SD of duration of symptoms of the two groups. No statistically significant difference was found between the two groups as p-value >0.05. DNS: dynamic neuromuscular stabilization

Demographic data analysis, conducted through independent t-tests, revealed no statistically significant distinctions between the dynamic neuromuscular stabilization (DNS) and Kegel exercise groups concerning age (Figure 2), height (Figure 3), weight (Figure 4), BMI (Figure 5), number of children (Figure 6), and duration of symptoms (Figure 7), as outlined in Table 2.

**Table 2:** Demographics of the study participants. P-value ≥0.05 shows no statistically significant difference between the DNS and the Kegel exercise group. BMI: body mass index; DNS: dynamic neuromuscular stabilization

Variables	Group	Range		Mean	SD	t-Value	p-Value
		Minimum	Maximum				
Age (years)	DNS	22	40	34.31	4.9	1.374	0.183
	Kegel	24	40	33.02	3.9		
Weight (kg)	DNS	40	78	61.08	8.1	-0.369	0.813
	Kegel	45	74	61.71	7.81		
Height (cm)	DNS	143	170	153.7	5.7	1.21	0.328
	Kegel	145	167	152.4	4.67		
BMI (kg/m^2^)	DNS	19.56	30.82	25.85	3.40	-0.996	0.342
	Kegel	21.08	30.8	26.51	2.8		
Number of deliveries	DNS	0	3	1.4	0.99	-0.339	0.745
	Kegel	0	3	1.53	0.86		
Duration of symptom (months)	DNS	6	48	20.04	10.25	0.36	0.973
	Kegel	6	60	19.95	12.73		

After the normality test using the Kolmogorov-Smirnov (KS) test, it was observed that the data deviated from a normal distribution. Subsequently, a square-root transformation was applied, and another normality test was executed. Following this transformation, variables such as perineometer values, EMG average, EMG peak, and EMG MVC exhibited normal distributions, as indicated in Table 3. These normally distributed variables underwent analysis using independent and paired t-tests to evaluate between-group and within-group differences, respectively. Conversely, the remaining variable, encompassing pressure biofeedback (PBU) readings, remains non-normally distributed (Table 3). As a result, Mann-Whitney U tests and Wilcoxon sign rank tests were employed for between-group and within-group comparisons of this variable. The subsequent results are elaborated in Table 3 below.

**Table 3:** Results showing the values of the Kolmogorov-Smirnov test for various outcome measures after transforming into square root values. The test results indicated that the pressure biofeedback variable significantly deviated from a normal distribution (p<0.05), while the other variables, including perineometer value, EMG peak, EMG MVC, and EMG average, did not significantly depart from normality (p>0.05). *P-value <0.05 shows a statistically significant deviation from the normal distribution. EMG: electromyography; MVC: maximum voluntary contraction

Dependent variables	Sig. two-tailed
Perineometer value (mmHg)	0.66
EMG peak (µV)	0.154
EMG MVC (%)	0.194
EMG average (µV)	0.310
Pressure biofeedback (mmHg)	<0.001*

Strength of the pelvic floor using perineometer

Initially, no significant difference existed between the two groups at baseline (p=0.065). However, at 12 weeks, a statistically significant group × time interaction was observed (t=8.56, p=0.009). Remarkably, the DNS group exhibited a substantial increase in PFM strength compared to the Kegel exercise group (Table 4). When comparing each group individually from baseline to 12 weeks, significant improvements were noted in both groups (DNS group: t = -19.74, p<0.001; Kegel exercise group: t = -12.82, p<0.001) (Table 5).

**Table 4:** Results showing the between-group comparison (independent t test) of perineometer and EMG values between DNS and Kegel exercise group at baseline and at 12 weeks of intervention. Mean±SD values of perineometer (mmHg) and EMG (peak, MVC, average) for DNS and Kegel exercise group at baseline and at 12 weeks of intervention. *P-value < 0.05 shows statistically significant difference between the two groups. EMG: electromyography; MVC: maximum voluntary contraction; DNS: dynamic neuromuscular stabilization

Outcome		Measurements	Group		t-Value	p-Value
			DNS (mean±SD)	Kegel (mean±​​​​​​​SD)		
Perineometer (mmHg)		Baseline	9.11±​​​​​​​2.71	9.02±​​​​​​​4.58	-2.02	0.065
		12 weeks	22.77±​​​​​​​4.3	14.21±​​​​​​​4.60	8.56	0.009*
EMG	Peak (µV)	Baseline	68.46±​​​​​​​43.11	76.41±​​​​​​​17.2	0.857	0.496
		12 weeks	150.52±​​​​​​​90.7	94.035±​​​​​​​33.6	2.5	0.001*
	MVC (%)	Baseline	47.05±​​​​​​​20.8	45.266±​​​​​​​21.72	0.469	0.707
		12 weeks	82.62±​​​​​​​38.8	59.133±​​​​​​​16.92	4.31	0.005*
	Average (µV)	Baseline	39.7±​​​​​​​19.08	41.02±​​​​​​​16.06	-0.678	0.445
		12 weeks	84.70±​​​​​​​37.4	58.08±​​​​​​​18.72	4.4	0.005*

**Table 5:** Results showing within group comparison (paired t-test) for perineometer and EMG values of DNS and Kegel exercise group at baseline and at 12 weeks of intervention. Mean±SD values of perineometer, EMG (peak, MVC, average) at baseline and at 12 weeks of intervention. *P-value < 0.05 shows statistically significant difference between baseline and at 12 weeks of measurement for various outcome measures. EMG: electromyography; MVC: maximum voluntary contraction; DNS: dynamic neuromuscular stabilization

Outcome		Measurements	Group	
			DNS	Kegel
Perineometer (mmHg)		Baseline (mean±SD)	9.11±2.71	9.02±4.58
		12 weeks (mean±SD)	22.77±4.3	14.21±4.60
		t-Value	-19.74	-12.82
		p-Value	0.000*	0.000*
EMG	Peak (µV)	Baseline (mean±SD)	68.46±43.11	76.41±17.2
		12 weeks (mean±SD)	150.52±90.7	94.035±33.6
		t-Value	-11.68	-8.58
		p-Value	0.000*	0.001*
	MVC (%)	Baseline (mean±SD)	47.05±20.8	45.266±21.72
		12 weeks (mean±SD)	82.62±38.8	59.133±16.92
		t-Value	-6.8	-5.61
		p-Value	0.000*	0.000*
	Average (µV)	Baseline (mean±SD)	39.7±19.08	41.02±16.06
		12 weeks (mean±SD)	84.70±37.4	58.08±18.72
		t-Value	-7.7	-12.80
		p-Value	0.000*	0.000*

Electromyography of the pelvic floor muscles

All EMG data were automatically filtered by the built-in software of the Infinity Myotrac. At baseline, no statistically significant difference was found between the groups for EMG average, EMG peak, and EMG MVC with p-values of 0.445, 0.496, and 0.707, respectively. However, a statistically significant group × time interaction was observed at 12 weeks for EMG average, EMG peak, and EMG MVC (t_avg_=4.4, p_avg_=0.005; t_peak_=2.5, p_peak_=0.001; t_MVC_=4.31, p_MVC_=0.005) as depicted in Table 4. Significant improvements were noted in both groups for all three components (EMG average, EMG peak, and MVC) when compared from baseline to 12 weeks (DNS group: t_avg_=6.2, p_avg_=0.000; t_peak_=5.12, p_peak_=0.000; t_MVC_=2.5, p_MVC_=0.000; Kegel exercise group: t_avg_=9.6, p_avg_=0.000; t_peak_=3.7, p_peak_=0.001; t_MVC_=5.3, p_MVC_=0.000) (Table 5).

Activation of transverse abdominis muscles using pressure biofeedback unit

No statistically significant difference was found between the two groups at baseline (p=0.360). Furthermore, no statistically significant group × time interaction was observed at 12 weeks between the two groups (z = -0.620, p=0.951) (Table 6).

**Table 6:** Results showing the between-group comparison (Mann-Whitney U test) for PBU values between DNS and Kegel exercise group at baseline and at 12 weeks of intervention. Median±SD values of PBU at baseline and at 12 weeks. *P-value > 0.05 shows no statistically significant difference between the two groups. PBU: pressure biofeedback unit; DNS: dynamic neuromuscular stabilization

Outcome	Measurements	Group		Z-value	p-Value
		DNS (median±SD)	Kegel (median±SD)		
PBU (mmHg)	Baseline	68±2.68	68±2.31	-0.915	0.360
	12 weeks	66±3.07	66±3.01	-0.620	0.951

However, significant improvements in pressure biofeedback unit (PBU) readings were noted in both groups when compared to baseline (DNS group: z = -5.45, p=0.000; Kegel exercise group: z = -3.868, p=0.000). Results are detailed in Table 7.

**Table 7:** Results showing within-group comparison (Wilcoxon sign-rank test) for PBU values of DNS and Kegel exercise group at baseline and at 12 weeks of intervention. Median±SD values of PBU at baseline and at 12 weeks. *P-value < 0.05 shows statistically significant difference between baseline and at 12 weeks of measurement for PBU. PBU: pressure biofeedback unit; DNS: dynamic neuromuscular stabilization

Outcome	Measurements	Group	
		DNS	Kegel
PBU (mmHg)	Baseline (median±SD)	68±2.68	68±2.34
	12 weeks (median±SD)	66±3.07	66±3.01
	Z-value	-5.45	-3.868
	p-Value	0.000	0.000

Calculation of Cohen’s D and effect size “r”

In our study, Cohen's D values were as follows: 2.89 for perineometer, 0.945 for EMGavg, 0.7 for EMGpeak, and 0.94 for EMG MVC. These values indicate that all variables had Cohen's D values greater than 0.7, signifying a medium to large effect size. This suggests that females in the DNS group experienced a more effective treatment compared to the Kegel exercise group for all the variables. However, for PBU effect size “r,” i.e., point biserial correlation coefficient, was conducted due to the use of a non-parametric test. The value of “r” for PBU was reported to be 0.06. The results are presented in Table 8.

**Table 8:** Values of Cohen's D and effect size "r" for the variables. MVC: maximum voluntary contraction

Variables	Cohen’s D/effect size “r”	Values of Cohen’s D or “r”
Perineometer	Cohen’s D	2.89
EMG (peak)	Cohen’s D	0.7
EMG (MVC)	Cohen’s D	0.94
EMG (average)	Cohen’s D	0.945
Pressure biofeedback	Effect size “r”	0.06

## Discussion

This study was conducted to evaluate the efficacy of dynamic neuromuscular stabilization (DNS) in the treatment of SUI among female participants aged between 18-40 years, comparing its effectiveness with conventional Kegel exercises. Our findings indicate that DNS exercises yielded notable benefits, effectively addressing SUI by enhancing activation of the transverse abdominis and core musculature, thereby surpassing the impact of Kegel exercises with a substantial effect size.

The findings revealed a statistically significant improvement in PFM strength in both the DNS and Kegel exercise groups after a 12-week intervention period. However, the DNS group exhibited a more substantial increase in PFM strength compared to the Kegel exercise group. This outcome aligns with previous research suggesting that DNS exercises, which target not only the pelvic floor but also the integrated spinal stabilization system, may lead to enhanced neuromuscular activation and coordination within the core musculature. These results emphasize the importance of incorporating dynamic stabilization exercises, as seen in DNS, to achieve optimal pelvic floor muscle function.

The EMG data analysis demonstrated significant improvements in EMG average, EMG peak, and MVC in both groups from baseline to 12 weeks. This indicates enhanced activation and strength of the pelvic floor muscles in response to the respective exercise interventions. The statistically significant group × time interaction underscores the differential impact of DNS exercises compared to traditional Kegel exercises on EMG parameters. The DNS group showed more pronounced improvements in EMG measures, highlighting the efficacy of a comprehensive approach targeting not only the pelvic floor but also the entire core musculature.

While both the DNS and Kegel exercise groups demonstrated improvements in pressure biofeedback unit (PBU) readings, indicative of transverse abdominis muscle activation, our analysis did not reveal a statistically significant difference between the two groups at the 12-week assessment. This finding suggests that both intervention approaches-DNS exercises and Kegel exercises-effectively engage the transverse abdominis muscle, a key component of core stability and pelvic floor support.

The lack of significant difference between the groups implies that both interventions may be similarly effective in targeting transverse abdominis activation, despite their differing approaches to pelvic floor rehabilitation. This finding underscores the importance of considering alternative exercise modalities, such as DNS, alongside traditional methods like Kegel exercises, in the management of stress urinary incontinence.

However, it is important to interpret these results cautiously, as the effect size "r" for PBU indicated a small correlation coefficient. This suggests that the observed impact on transverse abdominis activation may be relatively modest compared to other outcome measures assessed in the study. While both groups demonstrated improvements, the magnitude of these improvements may not be substantial enough to detect a statistically significant difference between the groups.

At the 12-week mark, significant differences were observed between the DNS and Kegel exercise groups across all outcome measures, except for pressure biofeedback readings related to transverse abdominis activation. It is noteworthy that the Kegel exercise group also demonstrated statistically significant improvements at the 12-week assessment for PBU readings compared to baseline measurements, highlighting the efficacy of pelvic floor muscle training not only in treating SUI but also in enhancing transverse abdominis activation.

This observation underscores the interconnectedness of pelvic floor and core musculature, challenging previous assertions suggesting isolated functioning of these muscle groups by some authors [4,5,21]. The results suggest a more integrated neuromuscular response, emphasizing the importance of comprehensive approaches in pelvic floor rehabilitation that encompass both pelvic floor and core muscle training to optimize outcomes for females with SUI.

The rationale behind employing DNS for stress urinary incontinence lies in the anatomical interconnectedness of core muscles, including the diaphragm, transverse abdominis, pelvic floor, and multifidus [22]. Numerous studies have highlighted the integral role of the lumbopelvic stability system in maintaining continence, emphasizing the inseparable action of the transverse abdominis and pelvic floor muscles [4,23,24]. Increased lumbar lordosis and pelvic floor strength have also been identified as key factors associated with stress urinary incontinence [25,26]. Notably, the training of the diaphragm and transverse abdominis has shown promise in improving urinary incontinence symptoms [15,27]. In light of these findings, our study advocates for a comprehensive approach by utilizing DNS, which addresses the synergistic action of the entire core musculature for the treatment of SUI.

The study's anticipated timeline for observing improvements, set at 12 weeks, aligns with established consensus in muscle physiology [15]. Prior research has similarly demonstrated discernible advancements in pelvic floor strength within this timeframe, thereby validating our study's results and highlighting the potential for meaningful outcomes through relatively short-term interventions [15].

Limitations

It is important to acknowledge certain limitations that may influence the interpretation and generalizability of the findings. The stringent exclusion criteria, including continuous urinary leakage and specific medical conditions, may have excluded individuals with diverse clinical presentations, potentially impacting the external validity of the study. Furthermore, the single-blinded nature of the study, while necessary for experimental rigor, introduces the possibility of bias, as participants may have been aware of their group assignment, potentially influencing their adherence or reporting of outcomes.

Moreover, the absence of diaphragm assessment represents a gap in our evaluation of the entire core musculature and its potential impact on stress urinary incontinence. Another limitation lies in the lack of long-term follow-up. A more extended follow-up period could provide valuable insights into the durability and persistence of the observed improvements.

While objective measures, such as perineometer values and EMG data, were collected, the study lacked subjective measures assessing participants' personal experiences and perceptions of improvement. Incorporating patient-reported outcomes could offer a more comprehensive understanding of the impact of DNS on the quality of life and well-being of individuals dealing with stress urinary incontinence.

## Conclusions

This study compares Dynamic Neuromuscular Stabilization (DNS) exercises with traditional Kegel exercises for managing stress urinary incontinence (SUI). DNS exercises showed significant improvements in pelvic floor strength and core musculature, aligning with emerging evidence supporting comprehensive interventions beyond conventional methods. The study's strengths include rigorous methodology and highlighting the crucial role of the lumbopelvic stability system in urinary continence. Future research could explore long-term treatment effects, optimal exercise protocols, and patient preferences to further advance pelvic floor rehabilitation. Overall, this study underscores DNS as a promising intervention for improving urinary continence in females with SUI.
